# Symptoms of Autism Spectrum Disorder (ASD) in Individuals with Mucopolysaccharide Disease Type III (Sanfilippo Syndrome): A Systematic Review

**DOI:** 10.1007/s10803-017-3262-6

**Published:** 2017-08-30

**Authors:** C. Wolfenden, A. Wittkowski, D. J. Hare

**Affiliations:** 1 Division of Psychology and Mental Health, School of Health Sciences, The University of Manchester and Manchester Acedemic Health Science Centre, Oxford Road, Manchester, M13 9PL UK; 2 Greater Manchester Mental Health NHS Foundation Trust, Manchester, Greater Manchester UK; 3 School of Psychology, Cardiff University, Cardiff, CF10 3AT UK

**Keywords:** Mucopolysacchardosis, Lysosomal storage disorder, Developmental disorder, ASD, Speech/language delay, Behavioural difficulties

## Abstract

The prevalence of autism spectrum disorder (ASD) in many genetic disorders is well documented but not as yet in Mucopolysaccharidosis type III (MPS III). MPS III is a recessively inherited metabolic disorder and evidence suggests that symptoms of ASD present in MPS III. This systematic review examined the extant literature on the symptoms of ASD in MPS III and quality assessed a total of 16 studies. Results indicated that difficulties within speech, language and communication consistent with ASD were present in MPS III, whilst repetitive and restricted behaviours and interests were less widely reported. The presence of ASD-like symptoms can result in late diagnosis or misdiagnosis of MPS III and prevent opportunities for genetic counselling and the provision of treatments.

## Introduction

Mucopolysaccharide diease type III (MPS III) belongs to a group of seven rare inherited metabolic disorders characterised by the deficiency of one of the lysosomal enzymes catalysing the degradation of glucosaminoglycans (GAG) or mucopolysaccharides. This deficiency results in abnormal accumulation of GAG in the lysosomes which consequently results in cellular damage and multi-systemic disease. Mucopolysaccharide disease type III (MPS III or Sanfilippo syndrome) is the most common of the mucopolysaccharidoses (Miekle et al. 1999). Four subytpes of MPS III have been indentified with their underlying genotypes and biochemistry established: MPA IIIA, IIIB, IIC and IIID (Valstar et al. 2008). Subtype A is the most prevalent in the UK, subtype B is less common and subtypes C and D are rare (Cleary and Wraith 1993). Similar rates are documented internationally (Valstar et al. 2008), although subtype B is known to be more common in South East Europe (Heron et al. 2010). Clinically, there is very little difference between the subtypes, but subtype A is known to follow a more severe course (Van De Kamp et al. 1981) and subtype C a more attenuated course (Ruijter et al. 2008).

Evidence suggests that many children with MPS III present with symptoms of Autism Spectrum Dmisdiagnisorder (ASD), such as language delay (Buhrman et al. 2013) and impaired social communication (e.g., Shapiro et al. 2016). According to Rumsey et al. (2014), symptoms of ASD are acquired (i.e., symptoms emerge at a later age following an otherwise typical development initially) in MPS III, suggestive of an atypical profile of ASD, as opposed to idiopathic ASD. The presentation of ASD-like symptoms has resulted in children with MPS III being misdiagnosed (‘misdiagnosis’- a term used by studies to describe an instance whereby ASD or another neurodevelopmental diagnosis has masqueraded a diagnosis of MPS III) with ASD and late diagnoses of MPS III (Wijburg et al. 2013). This has implications for genetic counselling and forestalls possible interventions for MPS III (Deshpande and Sathe 2015).

Previous reviews have identified the prevalence of ASD in a range of genetic syndromes (Richards et al. 2016) and demonstrated significant associations between ASD and other genetic developmental disorders (Moss and Howlin 2009). Previous reviews have identified the prevalence of ASD in a range of genetic syndromes (Richards et al. 2016) and demonstrated significant associations between ASD and other genetic developmental disorders (Moss and Howlin 2009). In their review of 42 studies of other genetic disorders, including Fragile X syndrome, Rett syndrome, Down syndrome, Tuberous Sclerosis Complex and phenylketonuria, Moss and Howlin (2009) indicated that symptoms of ASD are signficantly more likely to occur in individuals with such disorders than in the general population. Such symptoms may be present in the absence of a formal diagnosis of ASD and this supports the distinction between syndromic and non-syndromic variants of ASD. Understanding the overlap between ASD and genetic syndromes could potentially enable the genetic and biological pathways that underlie idiopathic ASD to be identified.

Despite the reviews identified above, no review to date has focused on the mucopolysaccharidoses. Although Wijburg et al. (2013) summarised the misdiagnosis of MPS III as ASD, to date, there has been no systematic review of the symptoms of ASD in MPS III. Consequently, this review aimed to (a) identify the extant literature on the symptoms of ASD in individuals with MPS III, (b) identify which symptoms are observed and (c) identify any common implications of ASD-like symptoms and (d) assess the quality of included studies.

## Method

A systematic search was conducted using Ovid to review five databases from inception to February 19th, 2017, namely  PsycInfo, Embase, Medline, Global Health and Health and Psychosocial Instruments. In addition, the references of included studies were hand-searched for relevant articles.

### Search Terms

**Fig. 1:**
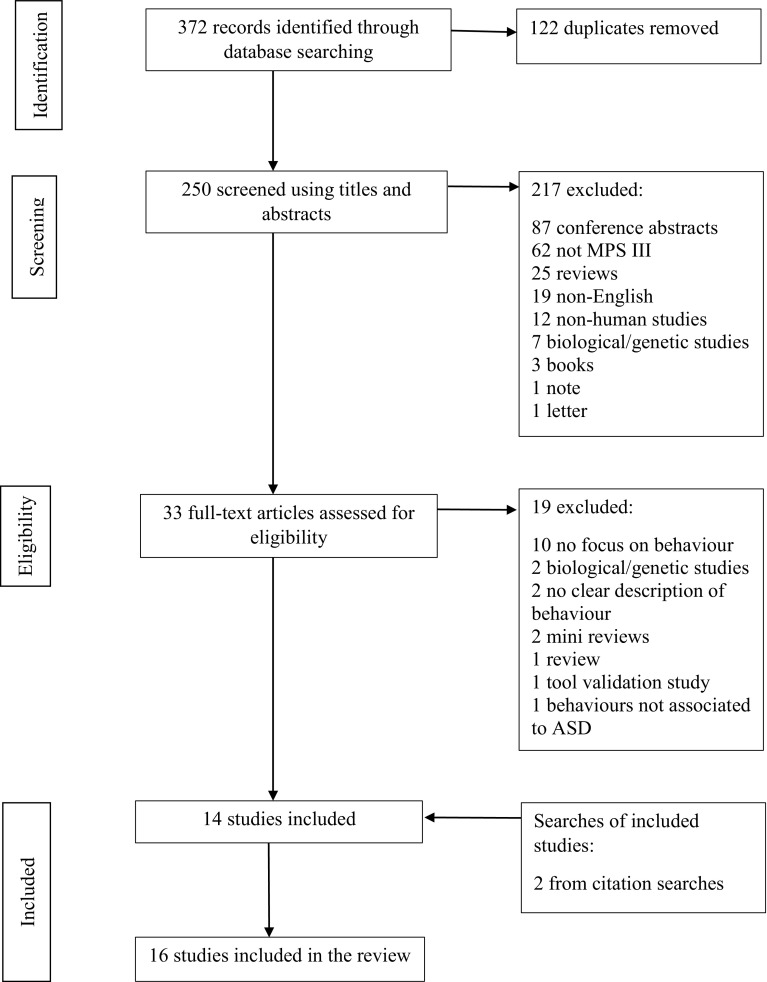
Flowchart demonstrating literature review process, Mucopolysaccharide disease (MPS), Autism spectrum disorder (ASD)

Search terms included ASD OR Autism OR Autis* OR pervasive developmental disorder OR communication difficulties OR social difficulties OR language delay OR speech delay OR delay OR behaviour problems OR behavioural problems OR restricted behaviour OR repetitive behaviour AND “Sanfilippo Syndrome OR Mucopolysaccharidosis OR Mucopolysaccharide disorder OR Mucopolysaccharide disease OR Mucopolysaccharide disease type III”. The search process, based on Preferred Reporting Items for Systematic Reviews and Meta-Analyses guidelines (PRISMA) (Moher et al. 2009) is outlined in Fig. 1.

### Inclusion and Exclusion Criteria

Inclusion criteria for the current review were studies published in English, which focused on MPS III and reported symptoms consistent with ASD (e.g., language or speech delay, communication difficulties, social difficulties, repetitive or restricted behaviour), whilst the following exclusion criteria were applied: non-English language, reviews and mini-reviews, studies which focused on other types of mucopolysaccharidoses, reports of behaviours inconsistent with ASD or a lack of detail surrounding behaviour and biological or genetic studies, i.e. reports on the biological rather than behavioural aspects of MPS III.

### Quality Assessment Tool

The *Quality Assessment Tool for Studies with Diverse Designs* (QATSDD) was selected (Sirriyeh et al. 2011), because it has good reliability and validity (Fenton et al. 2015) and because the methodologies used in these studies were expected to be diverse. Each of the 16 QATSDD items is rated on a 4-point-scale from “not at all” (0) to “complete” (3) and from which an overall quality rating of 0–42 can be computed. Consistent with the tool guidance (Sirriyeh et al. 2011), percentage scores were calculated (e.g., the total score was divided by the maximum potential score and multiplied by 100) and reported, with studies scoring over 75% considered to be of “high” quality, those between 50–75% “good”, 25–50% “moderate” and below 25% as “poor”. The first author (CW) and a peer colleague, independent to the study team, collaboratively rated all studies.

## Results

### Selection of Studies

A total of 16 studies (see Table 1) was identified for inclusion in the current review that reported on a total of 620 participants with diagnoses of MPS III. One study (Paper 15) involved two control groups, MPS I-Hurler Syndrome with eight participants and MPS IIIA with nine participants. Of the 620 participants with MPS III (see Table 2), 286 had confirmed sub-type A (MPS IIIA), 124 sub-type B (MPS IIIB), 56 had sub-type C (MPS IIIC), nine had sub-type D (MPS IIID) and 145 did not specify which sub-type. Four of the studies originated from the USA (Papers 2, 9, 11 and 15). Case report studies (Papers 3, 7, 9, 12, 13, 14 and 16) originated from Saudi Arabia, the Netherlands, Poland, Israel, India and Turkey, whereas cross-sectional studies (Papers 1, 2, 4, 5, 6, 8 and 10) originated from Denmark, the UK, the Netherlands, Sweden, France and Spain.

**Table 1:** Included papers presented in chronological order

Paper number	Author and date	Site of study	Design
1	Van de Kamp et al. (1981)	Denmark	Cross-sectional
2	Nidiffer and Kelly (1983)	America	Cross-sectional
3	Ozand et al. (1994)	Saudi Arabia	Case report
4	Bax and Colville (1995)	United Kingdom	Cross-sectional
5	Moog et al. (2007)	Netherlands	Cross-sectional
6	Malm and Mansson (2010)	Sweden	Cross-sectional
7	Verhoeven et al. (2010)	Netherlands	Case report
8	Heron et al. (2010)	France	Cross-sectional
9	Brady et al. (2013)	America	Case report
10	Delgadillo et al. (2013)	Spain	Cross-sectional
11	Rumsey et al. (2014)	America	Case series
12	Krawiec et al. (2014)	Poland	Case report
13	Sharkia et al. (2014)	Israel	Case report
14	Deshpande and Sathe (2015)	India	Case report
15	Shapiro et al. (2016)	America	Case control
16	Kartal (2016)	Turkey	Case report

**Table 2:** Reported demographics, symptoms of ASD and findings

Paper number	Sample size and sub-type of MPS III	Age and demographics	Symptoms of ASD	Assessment method	Reference to ASD or diagnosis of ASD	Findings	Quality rating score
1	73 A—36 B—23 C—14	41 of the 73 were from sibships 18 participants deceased 55 participants alive	Slow and poor speech development. (pre-age 3.5 years) Some never learned to speak Aggression in stressful situations	Review of medical records No details of formal assessments	Reports symptoms but no reference to ASD	Recognition of variability at the clinical level is of utmost importance for disease recognition, prognosis and genetic counselling	31% (Moderate)
2	30*	Mean age 10 years 16 female 14 male	Deterioration of communication skills Language problems seen at mean age 5.6 years Self-stimulatory seen at mean age 3. 8 years Peer difficulties seen at mean age 3.9 years Loss of social/adaptive behaviour (age 5 years)	Questionnaires based on: Bayley Scale of Infant Development, Stanford-Binet Intelligence Scale, Vineland Social Maturity Scale	Reports symptoms but no reference to ASD	Late diagnosis owing to focus on problematic behaviours resulted in no genetic counselling and thus further MPS III siblings	69% (Good)
3	1 D	Saudi Arabian female 7 years old	Self-stimulating behaviour Few words Variable eye contact (age 6) or no eye contact (age 7) Refers to “autistic behaviour” but no further detail (age 6)	Psychometric evaluation	Reference to “autistic like, self-stimulating behaviour” No formal diagnosis	Participant presented with acquired language disorder and failed to demonstrate the phenotype of MPS III	44% (Moderate)
4	106*	Mean age 8.4 years 40 male 56 female	Loss of language Abnormal reaction to invasion of social space	Questionnaire based on American Association of Mental Deficiency questionnaire and Rutter’s parent checklist	Reports symptoms but no reference to ASD	Some children with MPS III diagnosed late in life due to initial presentation as overactive retarded children by which time they had acquired one or more similarly affected siblings Greater emphasis needs to be placed on the need for earlier diagnosis	50% (Good)
5	20 B—20	Dutch Mean age of 43 years for alive participants 6 from consanguineous family 8 male (2 deceased) 12 female (6 deceased)	Delayed language and speech development Stereotypic speech Inability to keep up pace of peers No speech or unintelligible speech Hypersensitivity to touch and sensitive to temperature One participant never spoke Problems in making personal contacts	Observations of elderly residents with Intellectual Disability completed with care providers and/or parents Interviews with carers and family	Reports symptoms and refers to ASD in one case	In one patient metabolic screening was performed because of a suspicion of ASD IIIB may be diagnosed later in life due to a normal phenotype and because progression is slow	33% (Moderate)
6	22 A—15 B—1 C—5 type not determined—1	Ranged between 1.2 and 29 years 13 male (6 alive, 7 deceased) 9 female (4 alive, 5 deceased) 2 participants–immigrant children	No communication, few words Delayed early speech development in half of the sample Behavioural problems not defined	Parent report and professional impression	Behavioural problems suspected to be autism Not formally diagnosed Diagnoses of mental retardation by 6 or 7 years old	Hyperactivity and developmental delay could cover an early normal development and misdirect any suspicion of a progressive disease Behaviour problems suspected to be autism or ADHD	46% (Moderate)
7	1 B	White female 57 years old Non-consanguineous family	No peer interactions Repetitive Resisted physical contact Echolalia Hypersensitivity to physical contact	Unclear – not stated	Reference to “autistic like features” Not formally diagnosed	Focus on behavioural issues resulted in diagnosis at age 57 years	56% (Good)
8	French sample—128 A—87 B—18 C—17 D—6 UK sample—126 A—89 B—22 C—7 D—2 Not determined—6 Greek sample—20 B—16 C—3 Not determined—1	111 French 30 from multiplex families 126 British 40 from multiplex families 20 Greek, no multiplex families	France sample—language delay and ASD symptomology in: IIIA—29% IIB—19% IIIC—8% IIID—17% UK sample—85% had language delay and 66% ASD symptomology Greece sample—not stated	Parental impression and reports on questionnaires	Direct reference to ASD but not formally diagnosed	Early diagnosis should be followed by early treatment when available	50% (Good)
9	2 B—2	White Females 26 years & 31 years Non-consanguineous parents	Participant 1—Social immaturity Hyperactivity (preschool) Echolalia Participant 2—mute	Unclear—not stated	Reports symptoms but no reference to ASD Participant 1 was diagnosed with pervasive developmental disorder at age 8 years	MPS IIIB should be strongly considered in the differential diagnosis of patients with an early behavioural and psychiatric phenotype followed by progressive unexplained cognitive decline Late diagnosis of MPS IIIB at age 26 years	58% (Good)
10	55 A—34 B—11 C—10	Spanish 28 female 27 male Median age 13 years	Delayed speech in 85% by age 18 months Hyperactivity in 65% at mean age 3 years Lack of expressive language development	Questionnaire (not specified)	Reports symptoms but no reference to ASD	Diagnostic delay was common, particularly in patients with a slow progression or attenuated phenotypes, especially in MPS IIIC Need for early diagnosis for family genetic counselling e.g. gene therapy and enzyme replacement therapy	60% (Good)
11	21 A—21	14 male 7 female Mean age 4.5 years	Increased incidence of autistic like social behaviours emerging between 3 and 4 years Few or no words	ADOS	Reference to autistic like social behaviours 13/21 met criteria for formal diagnosis of ASD All children aged over 3.8 years met criteria and 2 aged under 3.8 years met criteria	A child exhibiting a lack of developmental gain or deceasing cognition coupled with autistic like social behaviours should raise a red flag for MPS IIIA in differential diagnosis Symptoms of autism are characteristic of disease progression Symptoms include a decrease in social communication Restricted interests and repetitive behaviours absent	62% (Good)
12	1 A	Male 5 years old Non-consanguineous parents	Lack of speech notable at age 5 years 1st words at age 18 months	Parent report	Refers to autism but no formal diagnosis	Misdiagnosis of autism can occur Screen for MPS III in children with behavioural abnormalities and developmental delay	47% (Moderate)
13	2 A—2	Palestinian Females 13 and 11 years old Consanguineous Palestinian family	“Autistic features” in patient 1 Speech delay in patient 2 and “autistic features” (age 18 months)	Parent and clinician impression	Refers to “autistic features” in both participants but no formal diagnoses	Misdiagnosis often occurs Autistic like features were found Patients should go directly for genetic testing if presenting with similar features	44% (Moderate)
14	1*	Female 7 years old	Reduced social interactions (age 6 years) Inability to understand speech (age 6 years) Repeatedly clapped hands and hit head (from age 2 years) Inability to understand and engage in routine social interactions (from age 2 years)	Parent and clinician impression	Refers to ASD but no formal diagnosis Participant initially diagnosed with pervasive developmental disorder	Autistic features are common with MPS IIIB Under diagnosis occurs Early diagnosis can improve chances of effectiveness of newer treatments MPS III should be considered in a child presenting with behavioural problems, who appears intellectually subnormal	36% (Moderate)
15	B—10	White 6 males 4 females Mean age 16 years	Impaired social communication Social/affective domain more affected than restricted or repetitive behaviours (over age 6 years)	ADOS	9/10 participants met criteria for ASD on ADOS	Phenotypic autistic like behaviours of both MPS IIIA and B may result in misdiagnosis MPS III B demonstrate symptoms associated with autism—impaired social communication Management techniques appropriate for ASD disorders might be appropriate for MPS III	74% (Good)
16	A	Turkish Male 7 years Non-consanguineous parents	Delayed speech Behavioural problems (not specified) aside from hyperactivity (from age 3 years)	Clinician impression Parent report	Reports symptoms but no reference to ASD	Initially diagnosed with ADHD Diagnostic process can be challenging and often protracted MPS III should be included in the differential diagnosis of developmental delay	22% (Poor)

*Indicates that subtype was not reported

The studies varied in their participant descriptions (see Table 2). All studies reported gender and age; however, only nine studies provided information surrounding ethnicity or nationality (Papers 3, 5, 6, 7, 8, 9, 12, 13 and 16). Half of the studies (Papers 1, 5, 7, 8, 9, 12, 13 and 16) provided additional data regarding the relationship between participants and their families. Paper 1 noted that 41 of 73 participants were from sibling relationships and Paper 8 reported on 70 of 111 participants who were from multiplex^1^ families. Consanguinity was another factor to which some studies referred to. Paper 5 highlighted that six of their 20 participants were from consanguineous families and Paper 13 described the case of a child from a consanguineous family. Whilst these two studies noted consanguinity, other studies (Papers 7, 9, 12 and 16) explicitly stated that participants were from non-consanguineous families.

Of the 16 studies, seven studies (Papers 1, 2, 4, 5, 6, 8 and 10) described and analysed cognitive, behavioural and motor difficulties within MPS III (Table 2). Seven of the studies were case reports (Papers 3, 7, 9. 12, 13, 14 and 16) which described presentations of MPS III and issues pertaining to its diagnosis and misdiagnosis. Paper 15 compared participants with MPS IIIB to participants with MPS IIIA and MPS IH (Hurler syndrome) and two studies (Papers 11 and 15) formally assessed symptoms of ASD in participants with MPS IIIA and MPS IIIB.

### Quality Ratings

Scores on the QATSDD ranged between 22 and 74% with a mean score of 49% (see Table 3).

**Table 3:** Quality ratings

	Item 1	Item 2	Item 3	Item 4	Item 5	Item 6	Item 7	Item 8	Item 9	Item 10	Item 11	Item 12	Item 13	Item 14	Item15	Item 16	Total raw score, average score (and % of highest possible score)
Paper 1	2	1	1	1	3	1	0	1	0	0	N/A	1	1	N/A	0	1	13, 1 (31)
Paper 2	3	3	2	1	2	2	3	3	2	3	N/A	2	1	N/A	0	2	29, 2 (69)
Paper 3	1	3	2	N/A	1	2	1	1	N/A	3	2	0	N/A	N/A	0	0	16, 1.3 (44)
Paper 4	3	3	2	1	2	1	1	1	1	2	N/A	2	0	N/A	0	2	21, 1.5 (50)
Paper 5	2	2	1	1	0	2	1	1	1	2	N/A	1	0	N/A	0	0	14, 1 (33)
Paper 6	2	3	2	2	2	2	1	1	0	2	2	2	0	0	0	1	22, 1.4 (46)
Paper 7	2	3	2	N/A	2	1	1	3	N/A	2	2	2	N/A	N/A	0	0	20, 1.6 (56)
Paper 8	3	3	2	2	3	1	0	2	0	2	N/A	2	0	N/A	0	1	21, 1.5 (50)
Paper 9	3	3	3	N/A	1	2	1	1	N/A	3	2	2	N/A	N/A	0	0	21, 1.8 (58)
Paper 10	3	3	1	2	2	2	1	3	1	2	N/A	3	1	N/A	0	1	25, 1.8 (60)
Paper 11	3	3	2	1	2	1	3	1	1	3	N/A	3	1	N/A	0	2	26, 1.9 (62)
Paper 12	2	3	3	N/A	1	1	0	1	N/A	2	2	1	N/A	N/A	0	1	17, 1.4 (47)
Paper 13	3	1	3	N/A	1	1	0	2	N/A	1	2	2	N/A	N/A	0	0	16, 1.3 (44)
Paper 14	2	2	3	N/A	1	2	0	1	N/A	0	1	1	N/A	N/A	0	0	13, 1 (36)
Paper 15	3	3	3	2	1	2	3	3	1	2	N/A	3	3	N/A	0	2	31, 2.2 (74)
Paper 16	1	1	1	N/A	1	1	0	1	N/A	0	1	1	N/A	N/A	0	0	8, 0.6 (22)

(0—not at all, 1—very slightly, 2—moderately, 3—complete)

Item 1—Explicit theoretical framework

Item 2—Statement of aims/objectives

Item 3—Clear description of research setting

Item 4—Evidence of sample size considered in terms of analysis

Item 5—Representative sample of target group of a reasonable size

Item 6—Description of data collection procedure

Item 7—Rationale for choice of data collection tools

Item 8—Detailed recruitment data

Item 9—Statistical assessment of reliability and validity (Quantitative only)

Item 10—Fit between stated research question and method of analysis

Item 11—Fit between stated research question and content of data collection (Qualitative)

Item 12—Ft between research question and method of analysis

Item 13—Good justification for analytical method selected

Item 14—Assessment of reliability of analytical process (Qualitative only)

Item 15—Evidence of service user involvement in design

Item 16—Strengths and limitations critically discussed

As previously mentioned, almost half of the studies (n = 7) reviewed were single case reports (Papers 3, 7, 9. 12, 13, 14 and 16); consequently, these were mostly rated as being of moderate methodological quality. In contrast, other studies were group-level studies (Papers 5, 11 and 15), with the three highest methodological quality studies coming from the USA (Papers 2, 11 and 15). Paper 16 obtained the lowest rating owing to a lack of information surrounding the choice of data collection, brief reference to the theoretical framework, justification for method and analysis and a lack of reference to the study’s strengths and limitations. In all 16 studies there was a lack of discussion surrounding the involvement of service users in their study designs.

Across the studies, seven obtained a moderate score for quality (Papers 1, 3, 5, 6, 12, 13 and 14) and eight were deemed to be of good quality (Papers 2, 4, 7, 8, 9, 10, 11 and 15). There was no observed relationship between quality and year of publication. Furthermore, as can be expected cross-sectional designs scored consistently better than the other designs reviewed. As this is the first review of the literature surrounding the symptoms of ASD in MPS III, all studies were retained to present a comprehensive picture of the available research.

### Symptoms of ASD

All of the 16 studies referred to behaviours characteristic of ASD, with ten studies (Papers 3, 5, 6, 7, 8, 11, 12, 13, 14 and 15) making a specific reference to ASD (Table 1). However, whilst these studies made specific reference to ASD, some case reports described observed behaviours in detail (Papers 9 and 14). In addition, some studies used the term ‘autistic features’ or ‘autistic like’ and failed to provide detailed descriptions (Papers 3,7 and 13). The lack of detailed descriptions was also noted in some of the cross-sectional studies (Papers 2, 4, 6, 8 and 10). For example, a relative strength of Paper 8 was its larger sample size when compared to the other studies, yet it did not describe symptoms of ASD in great detail.

When ASD-related behaviours and symptoms were specified and described in studies, these were categorised as:

*Speech, language and communication difficulties* Despite differing sample sizes, study designs and quality ratings, all 16 studies consistently reported difficulties with speech, language and communication (Table 2) with nine studies making explicit reference to this as a feature of ASD (Papers 3, 5, 7, 8, 11, 12, 13, 14 and 15). Difficulties in this domain included delayed language and speech development, limited vocabulary, no speech, echolalia, variable or no eye contact and impaired communication skills. The studies differed in reporting of age of onset of these behaviours, which were primarily reported as emerging after 18 months. When participants were assessed with a ‘gold-standard’ ASD assessment tool, the Autism Diagnostic Observation Schedule (ADOS) (Lord et al. 1999), they consistently met ADOS diagnostic criteria on communication domains (Papers 11 and 15).

*Repetitive and restricted behaviour* Two case reports reported repetitive and restricted behaviours. Paper 7 described a female participant (aged 57 years) with MPS IIIB who exhibited repetitive behaviour during adulthood, whereas Paper 14 described a 7-year-old girl (sub-type of MPS III not determined), presenting with repetitive hand clapping and head banging from the age of 2 years. However, neither study used standardised assessment tools. Two larger studies with sample sizes of 21 (Paper 11) and ten (Paper 15) utilised the ADOS, but reported little repetitive or restricted behaviour.

*Social difficulties* Ten studies observed social difficulties typical of an ASD presentation and these included aggression in social situations, peer difficulties and difficulties making personal contacts, social immaturity and impaired social interaction (Papers 1, 2, 3, 4, 5, 7, 8, 9, 11 and 14). As previously noted, not all studies adequately identified the age of the emergence of these behaviours, but those that did highlighted that these behaviours were evident from the age of 3 years. Four of the ten studies (Papers 2, 4, 11 and 15) used standardised assessment tools, such as the ADOS and Rutter’s parent checklist (Rutter et al. 1970), to formally assess for such behaviours, but the remaining six studies (a) failed to identify how behaviours were assessed and (b) failed to identify the assessment tool or (c) relied on professional or parent impression.

### Diagnosis of ASD

As previously mentioned, two studies (Papers 11 and 15) utilised the ADOS to assess behaviours of children with confirmed diagnoses of MPS IIIA and MPS IIIB; both studies were rated as having good methodological quality. Paper 11 concluded that 13 of the 21 children aged between 1.8 and 8.8 years with MPS IIIA met diagnostic criteria for ASD (as measured by module 1 of the ADOS) and that this was strongly associated with age; 11 children aged over 3.8 years met ADOS diagnostic criteria, compared with only two of ten children aged less than 3.8 years meeting ADOS diagnostic criteria. Paper 15 examined ASD in children with MPS IIIB and concluded that nine of their ten participants met ADOS criteria for ASD between the ages of 6 and 24 years. Both studies reported increased incidences of social/affective behaviour than restricted or repetitive behaviour. As part of their case reports, Paper 9 and Paper 15 described participants who presented with hyperactivity, poor social interactions and repetitive behaviour who consequently received diagnoses of pervasive developmental disorder prior to receiving a diagnosis of MPS III. In a large study of MPS III participants, Paper 8 noted “autism related symptoms” at time of diagnosis of MPS III in 66% of the UK MPS III population (n = 126) and in 76% of the French MPS III population (n = 128), but did not state whether any formal diagnoses of ASD had been made.

### Method of Behavioural Assessment

Studies that obtained higher scores on the QATSDD were noted to utilise formal questionnaires, standardised assessment tools and reported reliability and validity. Only two of the studies (Papers 11 and 15) assessed symptoms of ASD formally using the Autism Diagnostic Observation Schedule (ADOS) which was a strength of these studies. These studies more reliably and validly identified symptoms of ASD in individuals with MPS III. Paper 2 reported on the use of several validated and reliable assessment tools, including the Bayley Scale of Infant Development (Bayley 1969), the Stanford-Binet Intelligence Scale (Terman and Merrill 1973) and the Vineland Social Maturity Scale (Doll 1965). The findings of this paper suggested that children with MPS III begin to deteriorate cognitively between the ages of 3.5 and 6.5 years, lose language by age 8 years and demonstrate self-stimulatory behaviour and experience peer difficulties (Nidiffer and Kelly 1983). Whilst these tools do not specifically assess for ASD or repetitive behaviour and restricted interests, they do examine behaviours, such as language and social difficulties. A significant weakness of some studies (Papers 7 and 9) was failing to identify how behaviours were assessed at all, whereas other studies (Papers 6, 12, 13, 14 and 16), often of lesser quality (as assessed by the QATSDD) than the above-mentioned studies, referred to clinician and parent observation, impression and reports, which resulted in less valid conclusions and less robust study designs.

### Implications of Symptoms of ASD

#### Misdiagnosis

Seven of the 16 studies (Papers 3, 5, 6, 9, 12, 13 and 16) reported initial misdiagnosis (Table 2) including misdiagnoses of ASD, Attention Deficit Hyperactivity Disorder (ADHD), acquired language disorder and intellectual disability (reported as ‘mental retardation’). Such misdiagnoses were primarily observed in single-case reports and illustrated the phenomenological overlap between the behavioural phenotype of these disorders and MPS III.

#### Late Diagnosis

Seven of the 16 studies (Papers 2, 4, 5, 6, 7, 9 and 10) noted that a possible initial focus on problematic behaviour and developmental delay resulted in late diagnoses of MPS III. The larger sample sizes in five of these seven studies (Papers 2, 4, 5 and 6) suggest that this can be taken as a relatively reliable and consistent finding across presentations of MPS III. Three of the seven studies (Papers 5, 10 and 15) noted that sub-types MPS IIIB and IIIC were prone to late diagnosis owing to slow progression and attenuated phenotypes.

## Discussion

Previous reviews have identified the risk of symptoms of ASD in genetic syndromes (e.g., Moss and Howlin 2009; Richards et al. 2016). Despite the varying methodological quality of some of the studies included in this review, the current systematic review expands on Moss and Howlin’s (2009) review of seven other genetic syndromes by also noting the presence of symptoms of ASD in individuals with MPS III. Ten of the 16 studies reviewed made specific references to ASD in MPS III, referring broadly to “autistic features”, specifically to assessment of ASD and noting incidences of its diagnosis prior to diagnoses of MPS III. Speech, language and communication difficulties were consistently reported in all of the studies under review, repetitive and restricted behaviour less so. According to Rumsey et al. (2014), symptoms of ASD are acquired in MPS III and evidence from this review is suggestive of an atypical profile of ASD-like symptoms in MPS III yet cannot be concluded given the varying levels of detail reported in included studies, small sample sizes and varying methods of behavioural assessment. There was no consistent reporting of the onset of these speech, language and communication difficulties, but in line with a recent review that examined and reviewed the behaviours of 46 children with MPS IIIA (Buhrman et al. 2013), there was some indication that these difficulties were observed from 18 to 24 months.

Studies varied in their methodology surrounding the assessment of behaviours considered to be ASD and only two studies used a ‘gold-standard’ tool for assessing ASD, the ADOS. When this was used, 13 of 21 participants with MPS IIIA and nine of ten participants with MPS IIIB met ADOS diagnostic criteria for ASD. Similarly to Wijburg and colleagues (2013), this review highlighted the implications of misdiagnosis and late diagnosis of MPS III. Early diagnosis of MPS III can be challenging (Bodamer et al. 2014), partly as the result of the wide clinical variability in MPS III but also because clinicians tend to focus on the behavioural and developmental issues that are the initial presenting symptoms of MPS III.

### Clinical Implications

There is overlap between the behavioural phenotypes of both ASD and MPS III, particularly in the domains of speech, language, communication and social difficulties. When these behaviours occur alongside other physical or developmental abnormalities, clinicians should consider screening for MPS III to allow for early identification and diagnosis. In addition, clinicians should pay attention to the presence of sleep difficulties, especially complete reversals of day-night rhythms and impaired circadian functioning, facial dysmorphisms and recurrent ear, nose and throat infections (Mahon et al. 2014; Valstar et al. 2008). Benefits of earlier recognition and diagnosis of MPS III include genetic counselling for the family (Nidiffer and Kelly 1983), increased eligibility for effective treatments to take place (Wijburg et al. 2013) and potentially improved quality of life. Recognition of ASD-like symptoms, whether idiopathic or associated with genetic disorders of known aetiology, warrants the provision of tailored and evidenced behavioural support for individuals with MPS III, including interventions that support communication and social skills (see Hare 2015).

### Limitations

To ensure that all eligible and relevant studies were included in the current review, the initial search terms were deliberately kept broad. This strategy was successful in identifying 250 studies that were subsequently checked against the specific inclusion and exclusion criteria. As it was expected that the resultant selection would use a variety of methodologies, the quality assessment was undertaken using a measure (the QATSDD) specifically designed for assessing diverse designs. The QATSDD was useful in guiding both raters in the assessment of included papers but limitations of the tool were noted. The QATSDD does not include a quality assessment indicator for bias, and some indicators for the quality assessment lack detail, thus raters can apply them in different ways (Fenton et al. 2015). Collaborative quality assessment was useful in managing this because discrepancies could be discussed. To account for some of the limitations of the QATSDD, some adjustments were made to the calculation of the overall quality score to allow for better comparisons between studies, but this also means that these scores should be viewed with some caution.

Many of the studies included in this review had small sample sizes and lacked detail regarding symptoms and behaviours characteristic of ASD. Furthermore, almost half of the studies included were case reports and given the bias inherent in case study designs, readers are encouraged to consider all information and conclusions drawn from these studies carefully. Although this approach limits the strength of the conclusions drawn from the studies, it was necessary to include these because they are reflective of the current research and literature within MPS III.

Individuals with MPS III experience deteriorations in intellectual functioning (Grant et al. 2012) and there is an increased risk of co-occurring ASD in individuals with intellectual disability (Schieve et al. 2015). The studies cited in this review did not explicitly report on levels of intellectual functioning which should be an important consideration when assessing for and diagnosing ASD and consequently this should be considered a limitation of this review.

### Future Directions

A key limitation of many of the studies reviewed was the small sample sizes which is necessarily problematic for inferential statistical analysis, but also common in studies of rare disorders (De la Paz et al. 2010). However, this should not prevent research into rare disorders, such as MPS III, and sample sizes should be considered with regard to epidemiology of the syndrome. Further research could consider the use of a Bayesian approach (Howson and Urbach 2005) to statistical inferences in the study design of rare diseases (Billingham et al. 2011), because this enables information gathered from previous studies (particularly case studies) to contribute to estimation processes as opposed to the traditional hypothesis testing observed in larger samples.

All of the studies in the review made reference to behaviour that was typical of ASD but not all recognised this and some did not provide further details. Future research should detail more robustly the profile of symptoms of ASD in MPS III to understand whether it features as idiopathic ASD or simply symptoms of ASD. So far, the evidence suggests that the profile of ASD is largely compiled of speech, language, communication and social difficulties with little evidence of restricted or repetitive behaviour.

Only one of the 16 studies in this review (Shapiro et al. 2016) compared the assessment of symptoms of ASD in MPS III with a control group of another syndrome. The gold-standard assessments of symptoms of ASD should always include comparison to another syndrome to assess the degree of difficulty and comparisons to idiopathic ASD to evaluate the similarities and the differences in the profile of behaviour. Future research including this could improve our understanding of the psychological constructs associated to ASD in MPS III and their developmental trajectory.

## Conclusions

While the evidence base is neither large nor methodologically robust, this review finds evidence that symptoms of ASD are present in individuals with MPS III, specifically within the domains of speech, language and communication. Such symptoms can prevent and forestall clinical diagnosis of MPS III, resulting in reduced opportunities for genetic counselling and effective treatments. As MPS III is a rare disorder, the recommendations arising from this review are particularly important because they aim to support the growth of its emerging research literature to inform clinical practice. Understanding the development of ASD in MPS III could lead to the improved understanding of the neuropathology of MPS III and furthermore, a greater understanding of how the related emergence of both ASD and neurocognitive decline associated with MPS III could clarify disease progression and the neural substrate associated with both.

## Footnotes

1 A family in which a person diagnosed with a complex genetic disorder has a 1st- or 2nd-degree relative with the same diagnosis.
